# Identification of Immune-Related Hub Genes in Parkinson’s Disease

**DOI:** 10.3389/fgene.2022.914645

**Published:** 2022-07-22

**Authors:** Lin Chen, Yong Wang, Juan Huang, Binbin Hu, Wei Huang

**Affiliations:** ^1^ Department of Neurology, The Second Affiliated Hospital of Nanchang University, Nanchang, China; ^2^ Department of Oncology, The First Affiliated Hospital of Nanchang University, Nanchang, China

**Keywords:** Parkinson’s disease, weighted gene coexpression network analyses (WGCNA), LASSO, immune cell, hub genes

## Abstract

**Background:** Parkinson’s disease (PD) is a common, age-related, and progressive neurodegenerative disease. Growing evidence indicates that immune dysfunction plays an essential role in the pathogenic process of PD. The objective of this study was to explore potential immune-related hub genes and immune infiltration patterns of PD.

**Method:** The microarray expression data of human postmortem substantia nigra samples were downloaded from GSE7621, GSE20141, and GSE49036. Key module genes were screened *via* weighted gene coexpression network analysis, and immune-related genes were intersected to obtain immune-key genes. Functional enrichment analysis was performed on immune-key genes of PD. In addition to, immune infiltration analysis was applied by a single-sample gene set enrichment analysis algorithm to detect differential immune cell types in the substantia nigra between PD samples and control samples. Least absolute shrinkage and selection operator analysis was performed to further identify immune-related hub genes for PD. Receiver operating characteristic curve analysis of the immune-related hub genes was used to differentiate PD patients from healthy controls. Correlations between immune-related hub genes and differential immune cell types were assessed.

**Result:** Our findings identified four hub genes (*SLC18A2*, *L1CAM*, *S100A12*, and *CXCR4*) and seven immune cell types (neutrophils, T follicular helper cells, myeloid-derived suppressor cells, type 1 helper cells, immature B cells, immature dendritic cells, and CD56 bright natural killer cells). The area under the curve (AUC) value of the four-gene-combined model was 0.92. The AUC values of each immune-related hub gene (*SLC18A2*, *L1CAM*, *S100A12*, and *CXCR4*) were 0.81, 0.78, 0.78, and 0.76, respectively.

**Conclusion:** In conclusion, *SLC18A2*, *L1CAM*, *S100A12*, and *CXCR4* were identified as being associated with the pathogenesis of PD and should be further researched.

## Introduction

Parkinson’s disease (PD) is a progressive neurodegenerative disease. The condition is characterized by motor symptoms, comprising bradykinesia, resting tremor, rigidity, and nonmotor symptoms, including olfactory loss, autonomic dysfunction, depression, cognitive impairment, and insomnia. In 2016, more than 6 million people suffered PD worldwide, and the number of PD patients is estimated to increase with population age (GBD 2016 Parkinson’s Disease Collaborators, 2018). The major pathological features of PD are progressive loss of dopaminergic neurons in the substantia nigra and aberrant α-synuclein aggregation called Lewy bodies (Kalia and Lang, 2015). The exact etiology of sporadic PD remains unknown.

Growing evidence supports that immune dysfunction plays an essential role in the pathogenic process of PD. A nationwide epidemiological study from Sweden involving 310,522 patients with autoimmune disorders showed that 932 patients developed subsequent PD after follow-up; patients with an autoimmune disease had a 33% overall excess risk of PD (Li et al., 2012). A nested case–control study published in 2022 demonstrated that people with rheumatoid arthritis using immunosuppressant treatments were associated with a potentially decreased risk of developing PD (Paakinaho et al., 2022). Imaging of neuroinflammation with [^11^C] (R)-PK11195 PET showed that microglial activation is higher in PD patients than in healthy controls, confirming a link between neuroinflammation and the pathological process in PD (Gerhard et al., 2006). The levels of activated immune cells and proinflammatory cytokines are increased in the brain or cerebrospinal fluid of patients with PD (Mogi et al., 1996; Brodacki et al., 2008; Zhang et al., 2016; Schröder et al., 2018; Lin et al., 2019) and in the substantia nigra of the MPTP-induced PD animal model (Hébert et al., 2003). Hence, regulation of immune function might provide a potential therapeutic strategy to improve the prognosis of PD.

We focused on identifying potential immune-related hub genes and immune infiltration patterns of PD. The immune-related hub genes and immune infiltration patterns of PD were identified by bioinformatics analysis in this article. Weighted gene coexpression network analysis (WGCNA) was performed to obtain key module genes. A dataset of immune-related genes was acquired from an article (https://www.sciencedirect.com/science/article/pii/S2211124716317090), which is representative of 28 peripheral immune cell types. The immune-related genes and key module genes were intersected to obtain immune-key genes, and then, functional enrichment analysis was applied to immune-key genes of PD. In addition to, immune infiltration analysis was performed between PD samples and control samples using the single-sample gene set enrichment analysis (ssGSEA) algorithm to explore the differential immune cell types in the substantia nigra. Least absolute shrinkage and selection operator (LASSO) analysis was used to discover the immune-related hub genes, and receiver operating characteristic (ROC) curve analysis of immune-related hub genes was used to differentiate PD patients from healthy controls. The correlations between immune-related hub genes and differential immune cell types were analyzed. The expression levels of immune-related hub genes were verified in GSE20164. The immune-related hub genes and immune infiltration patterns of PD can be regarded as new therapeutic targets for PD.

## Materials and Methods

### Data Processing

**FIGURE 1:**
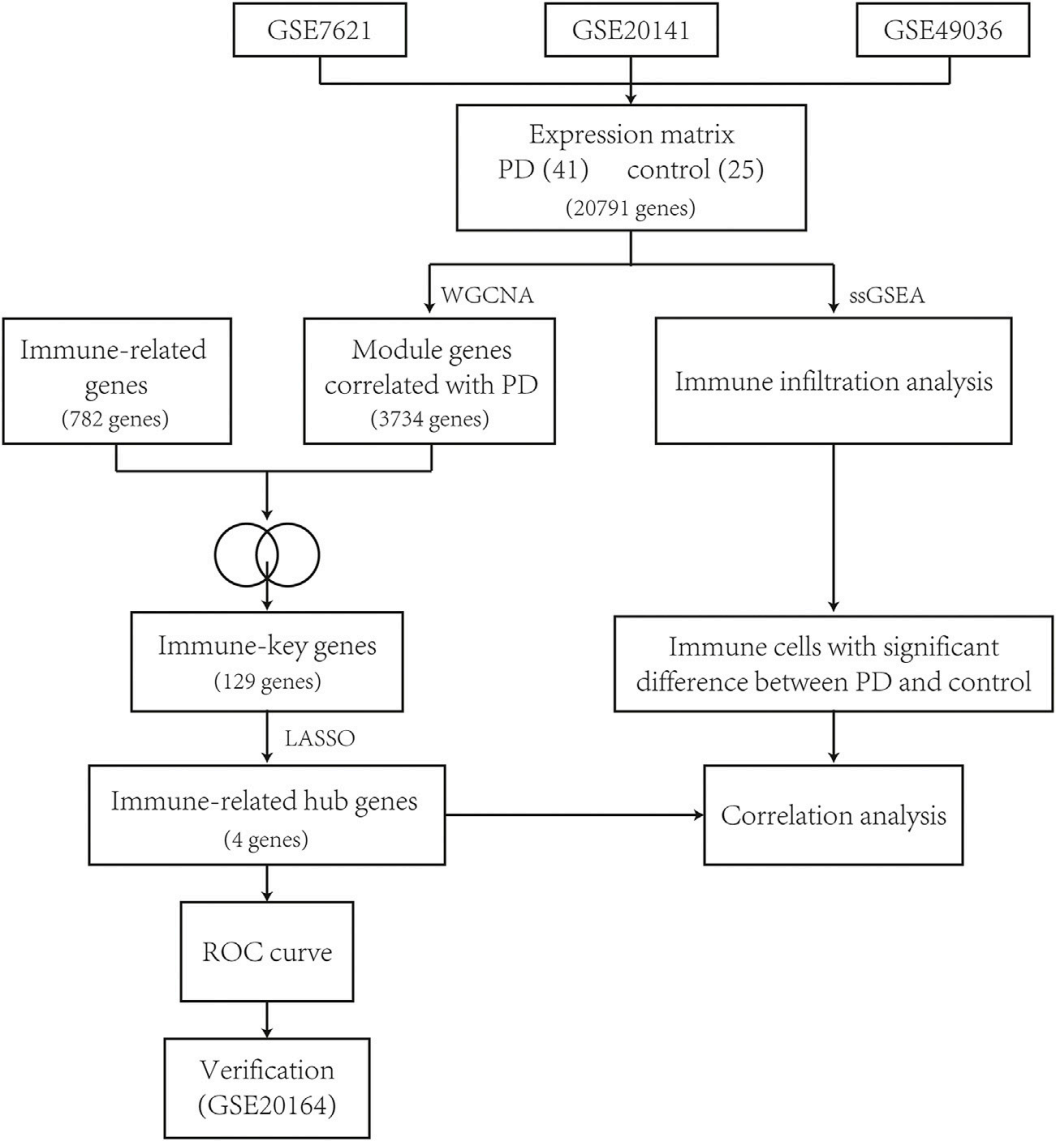
Flowchart of this study.

The workflow of this study is described as a flowchart in Figure 1.

The RNA microarray data of human postmortem substantia nigra samples were downloaded from the GSE7621, GSE20141, and GSE49036 datasets of the Gene Expression Omnibus (https://www.ncbi.nlm.nih.gov/geo/). These datasets were analyzed using Affymetrix Human Genome U133 Plus 2.0 Array on the platform of GPL570 (HG-U133_Plus_2). There were a total of 66 samples, including 41 samples from PD patients and 25 samples from healthy controls as a control group (Table 1). Using the ComBat function from the “sva” package of R software version 4.1.0, batch effects of the expression data were removed.

**TABLE 1:** The number of samples for PD and controls in included datasets.

Tissue	GSE	Platform	PD	Controls
Substantia nigra	GSE7621	GPL570	16	9
	GSE20141		10	8
	GSE49036		15	8

### Weighted Gene Coexpression Network Analysis

All genes (20,791 genes) of the datasets were analyzed using the “WGCNA” package. First, the variances of all genes were calculated and ranked from high to low, and genes with variance beyond quartiles of all variances were chosen for further analysis (5,198 genes). In a subsequent manner, we performed hierarchical cluster analysis of all samples to remove the abnormal samples. The Pearson correlation coefficient between every two genes was calculated to construct a similarity matrix. Then, we chose the soft thresholding power value from 1 to 20 based on the pickSoftThreshold function to build scale-free topology. We checked the scale-free topology based on the connection degrees k and p(k). *Via* dynamic tree cutting to construct coexpression modules, the highly correlated genes were classified into the same module, and each module contained at least 30 genes. The module eigengene (ME) value of each module was calculated. Furthermore, the correlation coefficient and *p* value between the ME value and phenotype of clinical traits (type of disease, PD vs. control) were calculated. When the *p* value was less than 0.05, the modules associated with PD were considered key modules. Module membership (MM) was the correlation between a gene and its module. Gene significance (GS) is the relationship between a gene and a clinical trait.

### Immune Infiltration Analysis

ssGSEA was used to quantify the infiltration levels of 28 immune cells by converting each sample’s gene expression profile into an immune gene set enrichment profile. Both the PD and control samples were fit into immune infiltration analysis *via* the ssGSEA algorithm using the “GSEA” R package to calculate the infiltration abundance of immune cells in the substantia nigra. The differential immune cells between the two groups were screened (*p* value < 0.05).

### Identification of Immune-Key Genes and Functional Enrichment Analysis

A widely recognized immune-related gene dataset of 28 peripheral immune cell types was used to investigate (Charoentong et al., 2017). This dataset included 782 immune-related genes. Immune-related genes and key module genes screened *via* WGCNA were intersected to obtain immune-key genes. Functional enrichment analysis was performed on immune-key genes to explore the potential biological implications. GO enrichment and KEGG pathway analysis were performed using the “clusterProfiler” and “GOplot” R packages. The BH method was used for *p* value adjustment in both GO enrichment and KEGG pathway analysis. The results of functional enrichment analysis were considered significantly enriched if the *p* adjust value < 0.05. In addition to, the top 5 GO terms and top 10 KEGG pathways are shown visually in the bubble chart.

### Identification of Immune-Related Hub Genes Using LASSO Logistic Regression

LASSO analysis was used to select the best features for high-dimensional data on account of its strong predictive value and low correlation. We used LASSO analysis to further identify immune-related hub genes for PD. The LASSO model was established using the “glmnet” R package, which could distinguish PD patients from controls. The expression levels of immune-key genes and clinical traits (type of disease, PD vs. control) were applied to build a LASSO logistic regression. ROC curve analysis of the immune-related hub genes was used to differentiate PD patients from healthy controls using the “pROC” R package. Furthermore, the correlations between immune-related hub genes and differential immune cell types were assessed.

### Validation of Hub Genes

To verify the expression difference of the immune-related hub genes between PD and controls, the GSE20164 dataset was used for validation. There were six PD patients and five controls in this dataset. The expression difference of hub genes in GSE20164 is shown with a boxplot using the draw_boxplot function “tinyarray” package. The Kruskal–Wallis test was used to compare the expression levels of hub genes between the PD and control samples. Statistical significance was set at *p* < 0.05.

## Results

### Identification of Gene Coexpression Modules

**FIGURE 2:**
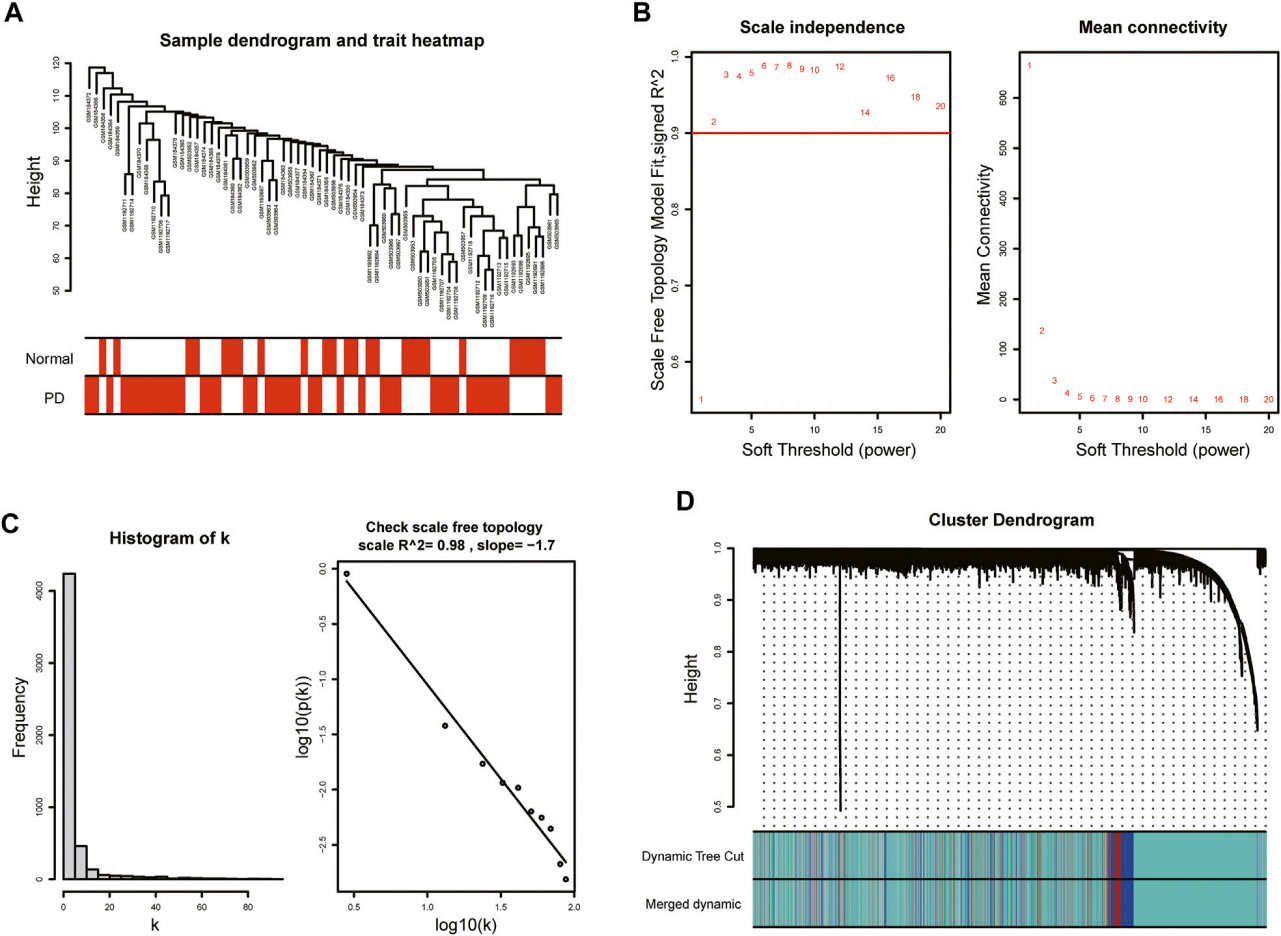
WGCNA of GSE7621, GSE20141, and GSE49036. **(A)** Sample dendrogram and trait heatmap. **(B)** The values of soft-threshold power based on scale independence and mean connectivity. The soft-threshold power was selected as 5 to satisfy the criteria of scale-free topology. **(C)** Check scale-free topology. The correlation coefficient of the connection degree k and p(k) was 0.98, indicating scale-free topology was constructed. **(D)** Cluster dendrogram of genes. Each color represented a module, and the gray module included the genes that could not be classified into any module.

With the purpose of more precise following analysis, 20,791 genes were obtained after removal of batch effects using ComBat, an empirical Bayes method (Supplementary Figure S1). The top 5,198 most varied genes from a total of 66 samples were selected to construct the coexpression network. All samples passed the cut-off line with a height of 120 followed by hierarchical clustering, and the clinical characteristic heatmap was drawn (Figure 2A). The soft thresholding power was selected as 5 based on the criteria of scale-free topology, with a scale-free *R* ^2^ value of 0.98 and a slope value of −1.7 (Figures 2B,C). Modules with divergences of less than 25% and fewer than 30 genes were merged into larger modules. Finally, four coexpression modules were determined (Figure 2D). The gray module consisting of non-coexpressed genes was considered an invalid module, which was excluded from the following analysis. There were 3,734 genes in the turquoise module, 521 genes in the blue module, 343 genes in the brown module, and 600 genes in the gray module.

### Calculation of Module-Trait Correlations

**FIGURE 3:**
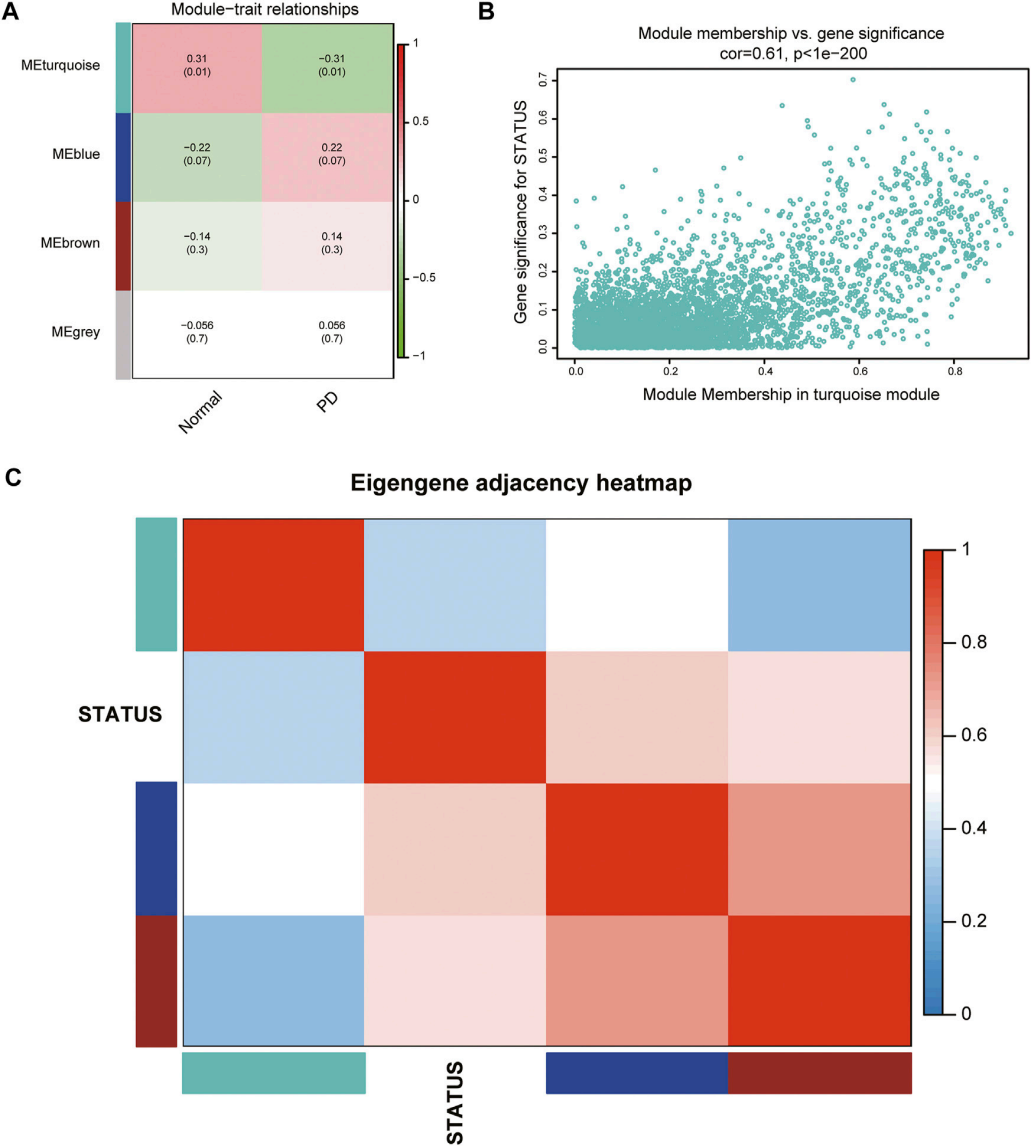
Identification of key modules associated with PD. **(A)** Heatmap of correlations between MEs and phenotype of clinical traits (type of disease). Red represented positive correlation and green represented negative correlation, and the corresponding *p* value was indicated in brackets. **(B)** GS and MM in the turquoise module. **(C)** Heatmap of the eigengene network representing the relationships among the modules and the clinical trait status.

According to the ME values of the obtained modules, the correlations between these modules and clinical traits (PD vs. control) were performed. The turquoise module revealed the highest correlation with PD (*r* = −0.31, *p* = 0.01) and was selected as the key module for further analysis (Figure 3A). The relationship between MM and GS was evaluated in the key modules, for which the correlation coefficient was 0.61 (*p* < 0.001), as depicted in Figure 3B. The heatmap of the eigengene network suggested that the turquoise module is highly related to the clinical trait status (Figure 3C).

### Immune Infiltration Analysis in PD

**FIGURE 4:**
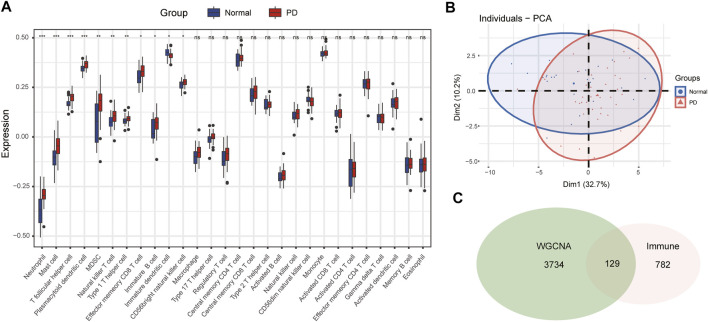
The immune cell infiltration analysis of substantia nigra between PD patients and healthy controls. **(A)** The landscape of immune cell infiltration in PD and healthy controls. There were eleven immune cells with significant difference (neutrophil, mast cell, T follicular helper cell, plasmacytoid dendritic cell, MDSC, natural killer T cell, type 1 helper cell, effector memory CD8 T cell, immature B cell, immature dendritic cell, and CD 56 bright natural killer cell). **(B)** Principal component analysis for immune cell infiltration in PD and healthy controls. **(C)** Venn diagram of genes screened *via* WGCNA and immune-related genes dataset. ns, *p* > 0.05, *p < 0.05, **p < 0.01, ***p < 0.001.

The infiltration abundance of 28 immune cell types in the substantia nigra between PD and control samples was calculated by the ssGSEA algorithm. The results revealed that neutrophils, mast cells, T follicular helper (Tfh) cells, plasmacytoid dendritic cells (DCs), myeloid-derived suppressor cells (MDSCs), natural killer (NK) T cells, type 1 helper (Th1) cells, effector memory CD8 T cells, immature B cells, immature DCs, and CD56 bright NK cells were significantly different in PD samples compared with control samples (Figure 4A). The infiltration abundance of immature DCs decreased in PD samples among differential immune cell types, whereas others increased in samples of PD compared to control samples. Furthermore, we performed principal component analysis (PCA) of immune cell infiltration among 66 substantia nigra samples. The PCA plot suggested that immune cell infiltration in the PD group was significantly different from that in the control group (Figure 4B).

### Identification of Immune-Key Genes and Functional Enrichment Analysis

**FIGURE 5:**
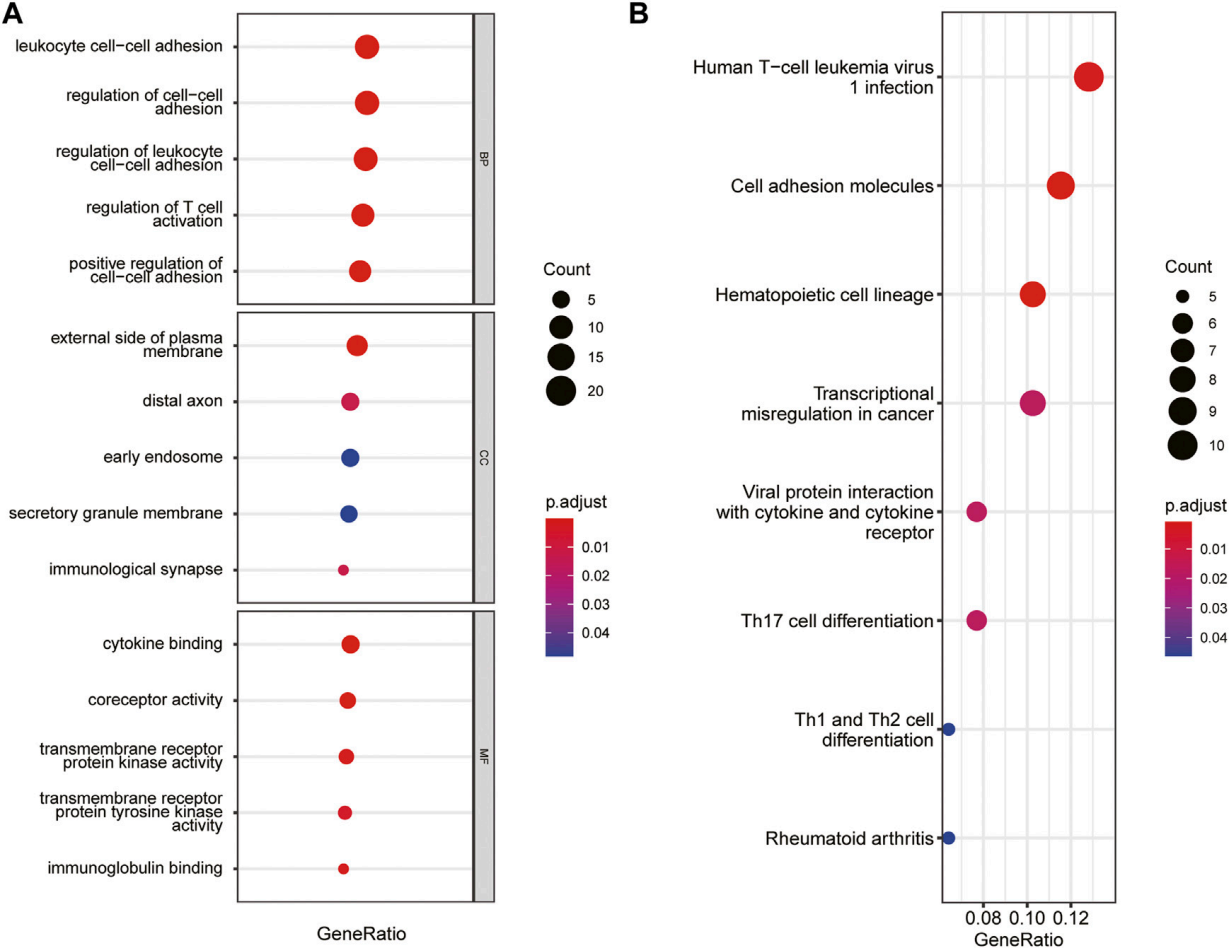
GO and KEGG pathways enrichment analysis of immune-key genes. **(A)** GO-BP, GO-CC, and GO-MF. **(B)** KEGG pathway. The color of the bubble represents the *p* value, and the size of the bubble represents the number of genes.

The immune-related gene dataset of 28 peripheral immune cell types was used for analysis, and 782 immune-related genes are shown in Supplementary dataset 1. A total of 129 immune-key genes were acquired *via* the overlap of immune-related genes and the key module genes (turquoise module), as displayed in Figure 4C. Then, GO enrichment and KEGG pathway enrichment analyses were performed on immune-key genes. GO enrichment analysis revealed that these genes were largely enriched in biological functions related to immune activities, such as regulation of leukocyte cell–cell adhesion and T-cell activation and cytokine binding (Figure 5A). KEGG pathway enrichment analysis showed that these genes were mainly involved in immune pathways, such as cell adhesion molecules and Th17-, Th1-, and Th2-cell differentiation (Figure 5B).

### Immune-Related Hub Gene Screening and Correlation Analysis of Hub Genes and Immune Cells

**FIGURE 6:**
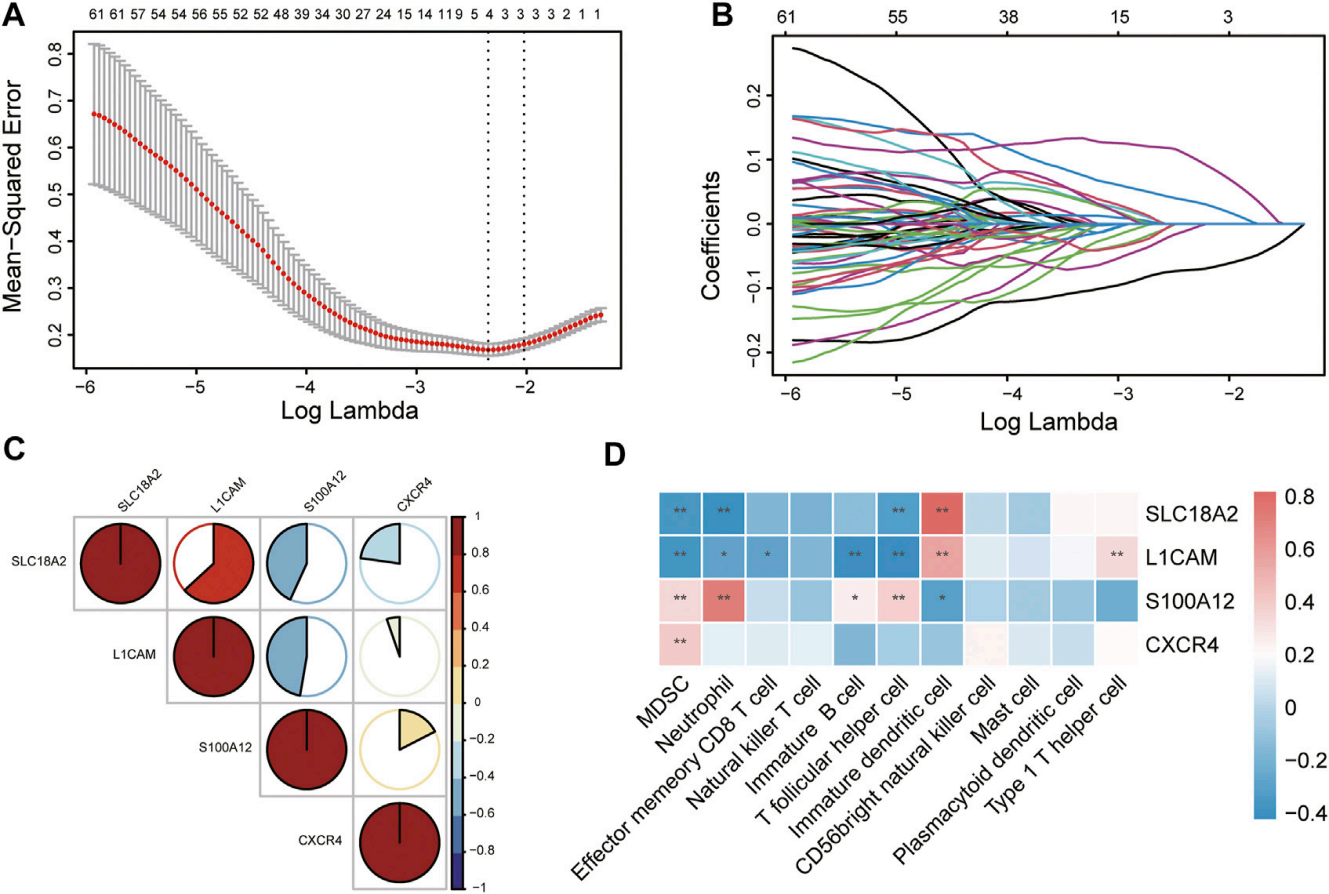
Identification of immune-related hub genes *via* LASSO model. **(A)** Tuning parameter (lambda) selection in the LASSO regression model. The vertical lines were drawn at the optimal values by minimum criteria and 1-SE criteria, and we selected minimum criteria to construct the model. **(B)** The LASSO coefficient profiles. **(C)** The relationship among immune-related hub genes. **(D)** The relationship between immune-related hub genes and immune cells. *p < 0.05, **p < 0.01.

To identify immune-related hub genes for PD, the expression levels of the immune-key genes and clinical traits (PD vs. control) in all samples were used to build the LASSO model (Figure 6A). A total of 129 immune-key genes were fit into LASSO logistic regression. According to the value of the lambda minimum criteria, four immune-related hub genes (*SLC18A2*, *L1CAM*, *S100A12*, and *CXCR4*) were identified to have nonzero regression coefficients (Figure 6B). The relationship among immune-related hub genes showed that *SLC18A2* was positively correlated with *L1CAM* and negatively correlated with *S100A12* and *CXCR4*; *L1CAM* was negatively correlated with *S100A12* and *CXCR4*; and *S100A12* was positively correlated with *CXCR4* (Figure 6C). The correlations between 4 immune-related hub genes and 11 differential immune cell types were assessed (Figure 6D). *SLC18A2* had a strong correlation with immature DCs (correlation coefficient = 0.82) and a moderate correlation with MDSCs, neutrophils, and Tfh cells (correlation coefficients were −0.35, −0.39, and −0.32, respectively). *L1CAM* had a moderate correlation with immature B cells, immature DCs, MDSCs, Tfh cells, and Th1 cells (correlation coefficients were −0.40, 0.56, −0.37, −0.42, and 0.34, respectively). There was a strong correlation between *S100A12* and neutrophils (correlation coefficient = 0.72) and a moderate relationship with immature DCs, MDSCs, and Tfh cells (correlation coefficients were −0.31, 0.35, and 0.38, respectively). *CXCR4* was moderately related to MDSCs (correlation coefficient = 0.40).

### ROC Curve Analysis of Immune-Related Hub Genes

**FIGURE 7:**
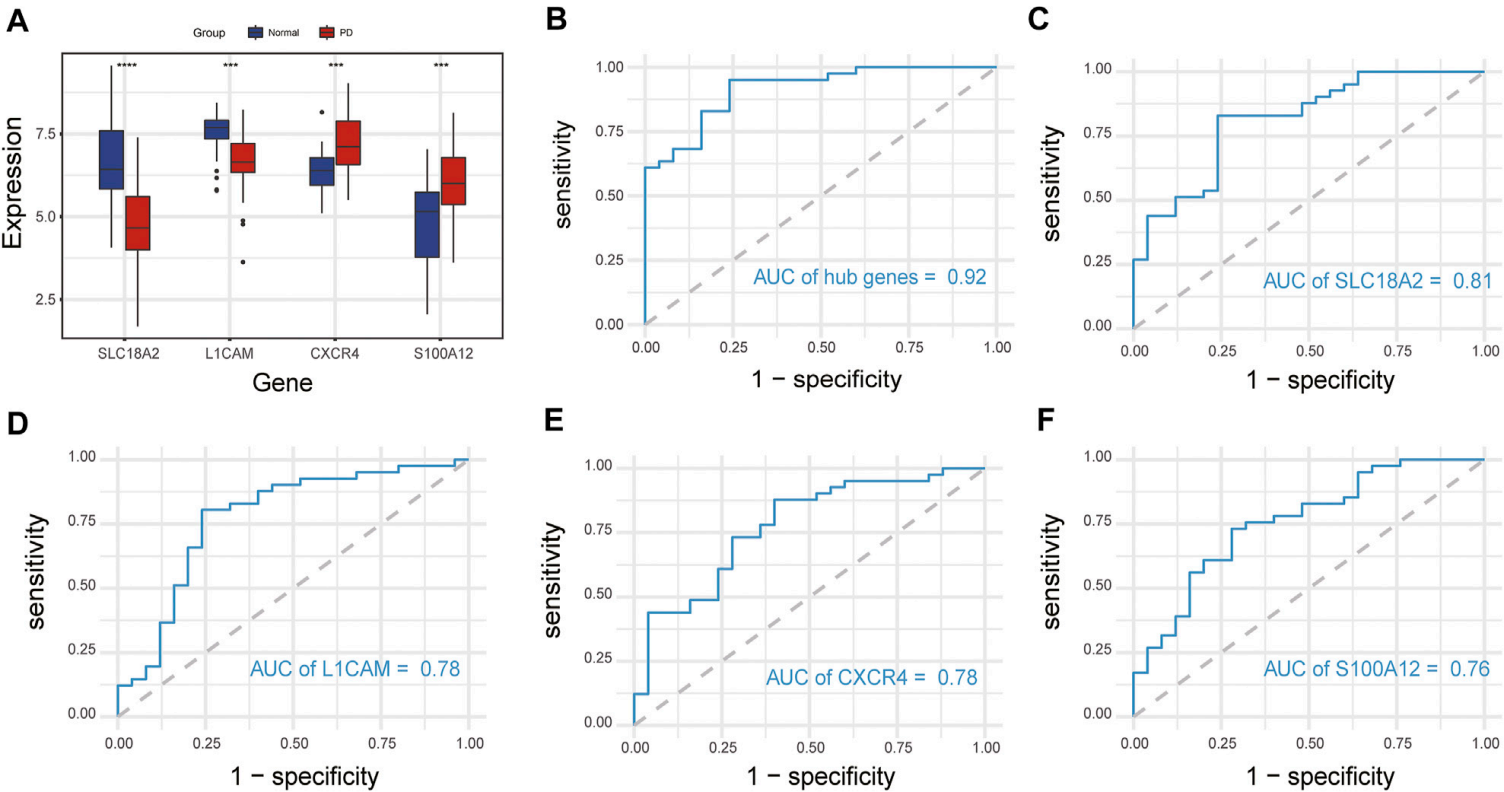
Expression of immune-related hub genes and ROC curve of immune-related hub genes. **(A)** Expression of hub genes in 41 PD and 25 healthy control samples. **(B)** The ROC curve of four immune-related hub genes. **(C–F)** The ROC curve of each immune-related hub gene. ns, *p* > 0.05, *p < 0.05, **p < 0.01, ***p < 0.001, ****p < 0.0001.

We compared the expression levels of immune-related hub genes in 66 samples, including 41 PD and 25 healthy control samples. The expression levels of *SLC18A2* and *L1CAM* were significantly downregulated, and *S100A12* and *CXCR4* were significantly upregulated in PD samples compared with control samples (Figure 7A). ROC curve analysis of the immune-related hub genes was performed to differentiate PD patients from healthy controls. As shown in Figure 7B, the area under the curve (AUC) value of the four-gene-combined model was 0.92. The AUC values of each immune-related hub gene (*SLC18A2*, *L1CAM*, *S100A12*, and *CXCR4*) were 0.81, 0.78, 0.78, and 0.76, respectively (Figures 7C–F).

### Validation of Immune-Related Hub Genes in GSE20164

**FIGURE 8:**
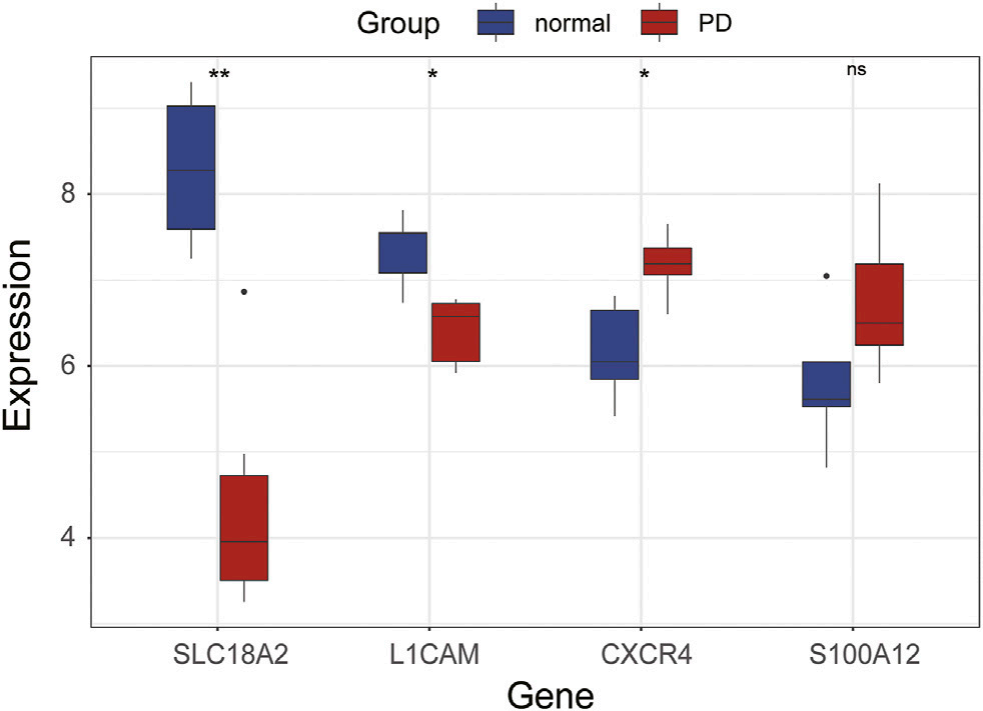
Expression of hub genes in GSE20164. ns, *p* > 0.05, *p < 0.05, **p < 0.01.

The expression levels of immune-related hub genes were verified in GSE20164. In GSE20164, there were six PD patients and five healthy controls. Figure 8 shows that the expression of *SLC18A2* and *L1CAM* was significantly downregulated and *CXCR4* was significantly upregulated in PD patients. There was no significant difference in *S100A12*.

## Discussion

PD was previously regarded as a movement disorder, but PD is now thought to be a multisystem disorder with notable neuroinflammation and immune dysfunction. Inflammatory manifestations that have been identified in PD patients include intestinal dysbiosis and inflammation, elevated circulating proinflammatory cytokine levels, innate and adaptive immune cell activation, blood–brain barrier breakdown allowing peripheral immune cell infiltration of the central nervous system, and chronic neuroinflammation (Tansey et al., 2022). In this study, *via* bioinformatics analysis, we explored the potential immune-related hub genes and immune infiltration patterns in the substantia nigra of PD patients, which provided new potential therapeutic biomarkers for PD.

Three datasets, GSE7621, GSE20141, and GSE49036, were included in our research. WGCNA established four distinct coexpression modules, among which the turquoise module was significantly related to the pathogenesis of PD. There were 3,734 genes in the turquoise module. The overlap of 782 immune-related genes and the turquoise module genes included 129 immune-key genes. GO and KEGG enrichment analyses demonstrated that immune-key genes focused on immune activities and pathways, including regulation of leukocyte cell–cell adhesion, regulation of T-cell activation, and Th17-, Th1-, and Th2-cell differentiation.

The immune infiltration analysis revealed eleven differential infiltrative immune cell types in the substantia nigra between PD and control samples: neutrophils, mast cells, Tfh cells, plasmacytoid DCs, MDSCs, NK T cells, Th1 cells, effector memory CD8 T cells, immature B cells, immature DCs, and CD56 bright NK cells. Based on LASSO logistic regression, four hub genes were finally screened: *SLC18A2*, *L1CAM*, *S100A12*, and *CXCR4*.

*SLC18A2* (also known as VMAT2, vesicular monoamine transporter 2) is expressed in both the peripheral and central nervous systems. VMAT2 takes up dopamine into intracellular vesicles. It was reported that dysfunction of VMAT2 proteins can result in cytoplasmic dopamine accumulation and lead to dopaminergic neuron death. Furthermore, VMAT2 mRNA levels were significantly reduced in PD patients versus healthy controls (Sala et al., 2010). *L1CAM* (cell adhesion molecule L1) is a transmembrane protein expressed in the brain and peripheral nerves that plays an important role in the development of the nervous system, cell adhesion, and synaptic plasticity. A study showed that the expression levels of *L1CAM* mRNA decreased in α-syn-treated cells (Sugeno et al., 2016). *S100A12* (S100 calcium binding protein A12) is an inflammation-associated protein expressed in neutrophils, macrophages, and epithelial cells and is related to Alzheimer’s disease (Shepherd et al., 2006), brain injury (Petrone et al., 2017), cancer, and so on. *CXCR4*, which is expressed in the central nervous system, is a chemokine receptor implicated in immune activity, microglia recruitment, and neurodevelopmental processes. Emerging evidence indicates that *CXCR4* is associated with neurodegenerative diseases, such as PD and progressive supranuclear palsy (Bonham et al., 2018). Postmortem brains showed that the expression levels of *CXCR4* in the substantia nigra and striatum of PD patients were higher than those in controls, accompanied by an increase in activated microglia (Shimoji et al., 2009). Moreover, *CXCR4* expression in the circulating mononuclear cells of PD patients is increased in comparison with that in controls (Bagheri et al., 2018).

In addition, we performed ROC curve analysis of immune-related hub genes to differentiate PD patients from healthy controls. The AUC value of the four-gene-combined model was 0.92. The AUC values of each immune-related hub gene were all above 0.75, indicating that these four hub genes might be signature genes of PD.

The correlations between 4 hub genes and 11 immune cells were evaluated. We discovered that neutrophils, Tfh cells, MDSCs, Th1 cells, immature B cells, immature DCs, and CD56 bright NK cells had moderate or strong correlations with hub genes, indicating that these immune cells are closely related to the pathogenesis of PD.

Oxidative injury is a characteristic feature of neurodegenerative diseases. Peripheral blood neutrophils are the predominant sources of reactive oxygen species. A study reported that oxidative stress levels in circulating neutrophils were higher in patients with PD than in controls. This research also revealed that mitochondrial mass and function were altered in neutrophils of patients with PD (Vitte et al., 2004). In another study, neutrophil counts were higher in PD patients than in healthy persons (Akıl et al., 2015).

Tfh cells, which are a subtype of CD4^+^ T cells, are relevant to B-cell differentiation, germinal center formation and humoral immune responses. Tfh cells in PD patients were significantly higher than those in controls (Zhao et al., 2020). In addition to, IL-4 was identified as a cytokine of Tfh cells and may participate in the degeneration of dopamine neurons in patients with PD (Bok et al., 2018).

MDSCs play an important role in the pathogenesis of cancers and inflammatory and autoimmune diseases. According to research findings, MDSCs inhibit inflammatory responses by suppressing CD4^+^ T-cell activation (Ostanin and Bhattacharya, 2013). The circulating MDSCs of newly diagnosed PD patients were higher than those of healthy controls and exhibited a pro-neuroinflammatory effect in PD (Chen et al., 2017).

Th1 cells are a subtype of CD4^+^ T cells. Flow cytometric analysis revealed that Th1 and regulatory T cells were increased in the midbrain tissue of a PD mouse model (Williams et al., 2021). The Th1 cytokines IFNγ and TNF have been shown to be increased in PD patient blood (Sulzer et al., 2017; Kustrimovic et al., 2018). A study published in 2009 suggested that CD4^+^ T and CD8^+^ T cells infiltrated specifically in the substantia nigra in the brains of PD patients (Brochard et al., 2009).

Peripheral immature B cells are an important member of the immune system, producing natural antibodies and regulating CD4^+^ T-cell responses (Lee et al., 2015). Dysfunction of immature B cells may contribute to autoimmune diseases. Emerging evidence suggests that B cells contribute to the pathogenesis of PD (Sabatino et al., 2019). B cells might be reduced in patients with PD (Bas et al., 2001; Stevens et al., 2012).

DCs are professional antigen-presenting cells related to the pathogenesis of neuroinflammation. DCs are recruited to the brain across the blood–brain barrier. A study revealed that immature DCs adhered to activated endothelial cells more avidly than mature DCs (Arjmandi et al., 2009).

NK cells are primarily divided into two major subsets (CD56 dim and CD56 bright) in humans. In the central nervous system, the majority of NK cells in cerebrospinal fluid are CD56 bright cell subsets (Han et al., 2014). CD56 bright NK cells modulate immune responses through cytokine production (Poli et al., 2009). Circulating NK cell counts increased in PD patients in contrast with non-PD controls (Mihara et al., 2008). In addition to, NK cells can internalize and degrade α-syn aggregates (Earls and Lee, 2020).

In this study, we discovered four immune-related hub genes (*SLC18A2*, *L1CAM*, *S100A12*, and *CXCR4*) and seven peripheral immune cell types (neutrophils, Tfh cells, MDSCs, Th1 cells, peripheral immature B cells, DCs, and NK cells) that are closely related to the pathogenesis of PD. The immune-related hub genes were mainly associated with biological pathways, including the dopaminergic neurotransmitter release cycle, developmental biology (such as axon guidance), and immune and inflammatory activity. Mounting evidence indicates that immune dysfunction is involved in the pathogenesis of PD. The physiological function of the axon guidance pathway includes neuronal network formation during central nervous system development and the maintenance and plasticity of neural synapses (Bae et al., 2021). Abnormal axon-guidance-molecule signaling can lead to loss of connectivity and eventually trigger PD (Lin et al., 2009; Tomiyama, 2011). In addition, peripheral immune cells infiltrate the brain, possibly because of blood–brain barrier breakdown, which may affect neuroinflammation in the central nervous system by modulating microglial and astrocyte functions. Microglia and astrocytes are central to neuronal function and health. Activation of microglia can transform neuroprotective astrocytes to neurotoxic astrocytes, inducing loss of neuroprotective function, and overactivated microglia can result in cerebral inflammation and neuronal injury (Tan et al., 2020).

There were several limitations in our study that should be acknowledged. First, our findings require *in vitro* experiments to verify the results. Second, samples from early-stage or prodromal PD patients and animal models are required to explore immune-related hub genes, which can provide diagnostic biomarkers and timely drug intervention. In future investigations, we will focus on the mechanism of *SLC18A2, L1CAM*, *S100A12*, and *CXCR4* and explore early diagnostic biomarkers in PD.

## Conclusion

Using bioinformatics analysis, immune-related hub genes in the substantia nigra of PD patients were identified. *SLC18A2*, *L1CAM*, *S100A12*, and *CXCR4* were regarded as candidate genes for further investigation. Our study provides immune-related genes involved in the pathogenesis of PD and promising therapeutic targets for PD.

## Data Availability

The original contributions presented in the study are included in the article/Supplementary Materials, and further inquiries can be directed to the corresponding author.
