# Supplementary material for: Identification of Immune-Related Hub Genes in Parkinson’s Disease
Source: Front Genet. 2022 Jul 22;13:914645. doi: 10.3389/fgene.2022.914645 (PMC9353688; doi:10.3389/fgene.2022.914645)
Supplement: Supplementary file 2 [file Table1.DOCX]

Table 1: The number of samples for PD and controls in included datasets

| Tissue | GSE | Platform | PD | Controls |
| --- | --- | --- | --- | --- |
| Substantia Nigra | GSE7621 | GPL570 | 16 | 9 |
|  | GSE20141 |  | 10 | 8 |
|  | GSE49036 |  | 15 | 8 |
