# Supplementary figures and images for: Identification of Immune-Related Hub Genes in Parkinson’s Disease
Source: Front Genet. 2022 Jul 22;13:914645. doi: 10.3389/fgene.2022.914645 (PMC9353688; doi:10.3389/fgene.2022.914645)

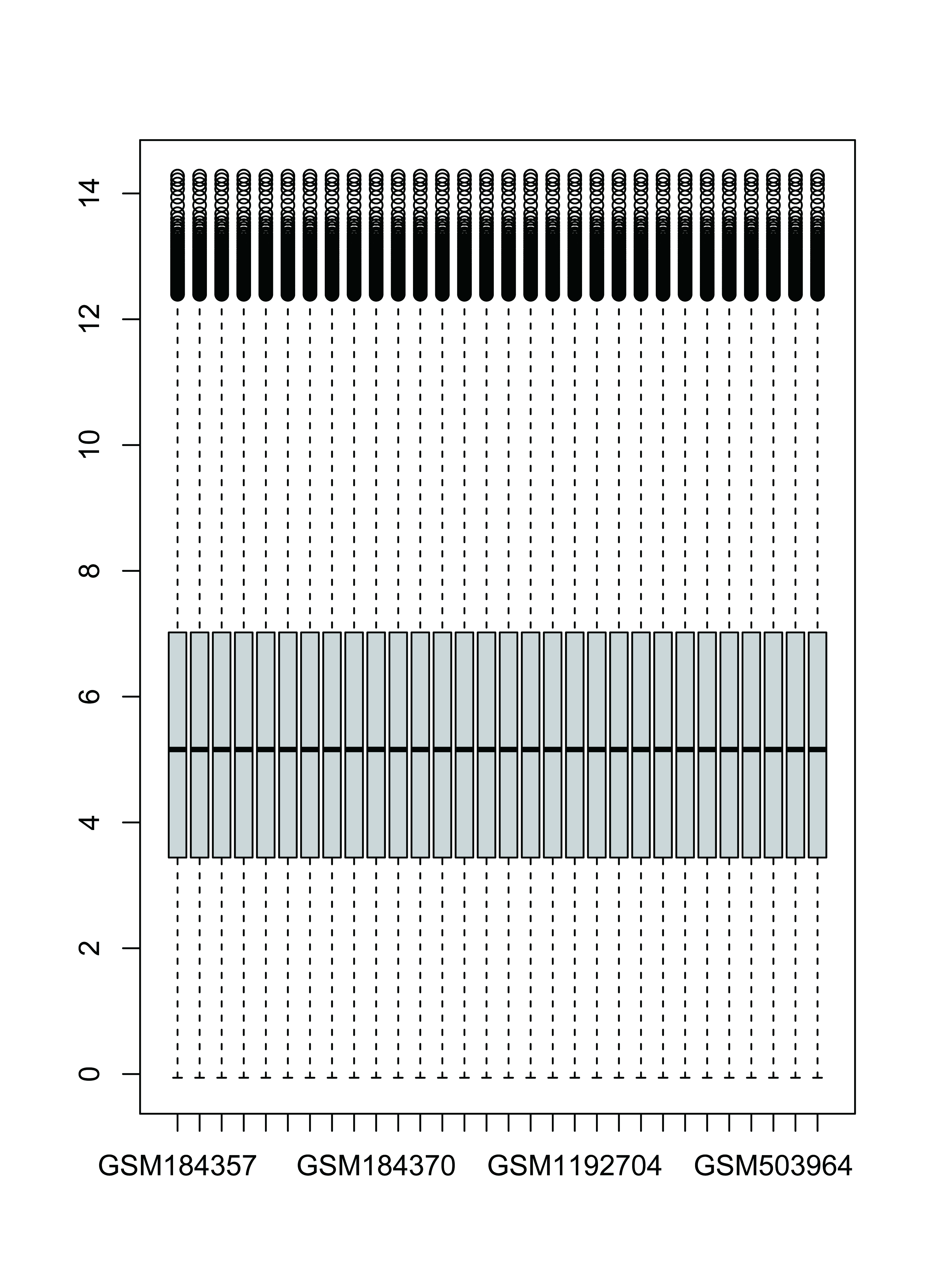

Supplement: Supplementary file 3 [file Image1.TIF]
