# Supplementary material for: Identification of Immune-Related Hub Genes in Parkinson’s Disease
Source: Front Genet. 2022 Jul 22;13:914645. doi: 10.3389/fgene.2022.914645 (PMC9353688; doi:10.3389/fgene.2022.914645)
Supplement: Supplementary file 4 [file Table3.DOCX]

**Figure legends**

Figure 1 Flow chart of this study.

Figure 2. WGCNA of GSE7621, GSE20141 and GSE49036. (A) Sample dendrogram and trait heatmap. (B) The values of soft threshold power based on scale independence and mean connectivity. The soft-threshold power was selected as 5 to satisfy the criteria of scale free topology. (C) Check scale free topology. The correlation coefficient of the connection degree k and p(k) was 0.98, indicating scale free topology was constructed. (D) Cluster dendrogram of genes. Each color represented a module, and the gray module included the genes that could not be classified into any module.

Figure 3. Identification of key modules associated with PD. (A) Heatmap of correlations between MEs and phenotype of clinical traits (type of disease). Red represented positive correlation and green represented negative correlation, and the corresponding p value was indicated in brackets. (B) GS and MM in the turquoise module. (C) Heatmap of Eigengene network representing the relationships among the modules and the clinical trait status.

Figure 4. The immune cell infiltration analysis of substantia nigra between PD patients and normal. (A) The landscape of immune cell infiltration in PD and normal. There were eleven immune cells with significant difference (Neutrophil, Mast cell, T follicular helper cell, Plasmacytoid dendritic cell, MDSC, Natural killer T cell, type 1 helper cell, Effector memeory CD8 T cell, Immature B cell, Immature dendritic cell and CD 56 bright natural killer cell). (B) Principal component analysis for immune cell infiltration in PD and normal. (C) Venn diagram of genes screened via WGCNA and immune-related genes dataset. ns, p > 0.05, * p < 0.05, ** p < 0.01, *** p < 0.001.

Figure 5. GO and KEGG pathways enrichment analysis of immune-key genes. (A) GO-BP, GO-CC, GO-MF. (B) KEGG pathway. The color of the bubble represents the p value, and the size of the bubble represents the number of genes.

Figure 6. Identification of immune-related hub genes via LASSO model. (A) Tuning parameter (lambda) selection in the LASSO regression model. The vertical lines were drawn at the optimal values by minimum criteria and 1-SE criteria, and we selected minimum criteria to construct the model. (B) The LASSO coefficient profiles. (C) The relationship among immune-related hub genes. (D) The relationship between immune-related hub genes and immune cells. * p < 0.05, ** p < 0.01.

Figure 7. Expression of immune-related hub genes and ROC curve of immune-related hub genes. (A) Expression of hub genes in 41 PD and 25 healthy control samples. (B) The ROC curve of four immune-related hub genes. (C-F) The ROC curve of each immune-related hub gene. ns, p > 0.05, * p < 0.05, ** p < 0.01, *** p < 0.001,**** p < 0.0001.

Figure 8. Expression of hub genes in GSE20164. ns, p > 0.05, * p < 0.05, ** p < 0.01.

**Supplementary Materials**

Supplementary 1. Supplementary Figure S1: expression data removed batch effects

Supplementary 2. Supplementary dataset 1: dataset of 782 immune-related genes
