# Supplementary material for: Identification of Immune-Related Hub Genes in Parkinson’s Disease
Source: Front Genet. 2022 Jul 22;13:914645. doi: 10.3389/fgene.2022.914645 (PMC9353688; doi:10.3389/fgene.2022.914645)
Supplement: Supplementary file 5 [file Table4.DOCX]

Supplementary information of R packages in the article:

1. sva -Leek JT, Johnson WE, Parker HS, Jaffe AE, Storey JD. The sva package for removing batch effects and other unwanted variation in high-throughput experiments. Bioinformatics. 2012 Mar 15;28(6):882-3. doi: 10.1093/bioinformatics/bts034 .
2. WGCNA - Langfelder P, Horvath S. WGCNA: an R package for weighted correlation network analysis. BMC Bioinformatics. 2008 Dec 29;9:559. doi: 10.1186/1471-2105-9-559.
3. GSVA - Hänzelmann S, Castelo R, Guinney J. GSVA: gene set variation analysis for microarray and RNA-seq data. BMC Bioinformatics. 2013 Jan 16;14:7. doi: 10.1186/1471-2105-14-7.
4. clusterProfiler - Yu G, Wang LG, Han Y, He QY. clusterProfiler: an R package for comparing biological themes among gene clusters. OMICS. 2012 May;16(5):284-7. doi: 10.1089/omi.2011.0118.
5. Goplot - Walter W, Sánchez-Cabo F, Ricote M. GOplot: an R package for visually combining expression data with functional analysis. Bioinformatics. 2015 Sep 1;31(17):2912-4. doi: 10.1093/bioinformatics/btv300.
6. glmnet - Friedman J, Hastie T, Tibshirani R. Regularization Paths for Generalized Linear Models via Coordinate Descent. J Stat Softw. 2010;33(1):1-22.
7. pROC - Robin X, Turck N, Hainard A, Tiberti N, Lisacek F, Sanchez JC, Müller M. pROC: an open-source package for R and S+ to analyze and compare ROC curves. BMC Bioinformatics. 2011 Mar 17;12:77. doi: 10.1186/1471-2105-12-77.
8. tinyarray - Xiaojie Sun. 2021 Nov 8. https://github.com/xjsun1221/tinyarray
